# Tag SNPs in complement receptor-1 contribute to the susceptibility to non-small cell lung cancer

**DOI:** 10.1186/1476-4598-13-56

**Published:** 2014-03-12

**Authors:** Xinfeng Yu, Juan Rao, Jia Lin, Zhi Zhang, Lei Cao, Xuemei Zhang

**Affiliations:** 1 Department of Pharmacology, School of Chemical Biology and Pharmaceutical Sciences, Capital Medical University, Beijing, China; 2 Department of Molecular Genetics, College of Life Science, Hebei United University, Tangshan 063000, China; 3 Department of Chemotherapy and Radiotherapy of Cancer, Tangshan Gongren Hospital, Tangshan, China

**Keywords:** CR1, Polymorphism, Tag SNPs, Lung cancer

## Abstract

**Background:**

Complement receptor 1 (CR1), the receptor for C3b/C4b complement peptides, plays a crucial role in carcinogenesis. However, the association of genetic variants of CR1 with susceptibility to lung cancer remains unexplored.

**Methods:**

This case-control study included 470 non-small cell lung cancer (NSCLC) patients and 470 cancer-free controls. Based on the Chinese population data from HapMap database, we used Haploview 4.2 program to select candidate tag SNPs. Odds ratios (ORs) and 95% confidence intervals (CIs) were computed by logistic regression to evaluate the association of each tag SNP with NSCLC.

**Results:**

Multivariate regression analysis indicated that the rs7525160 CC genotype was associated with an increased risk of developing NSCLC (OR = 1.52, 95% CI = 1.02-2.28; *P* = 0.028) compared with the GG genotype. When stratified by smoking status, the risk of NSCLC was associated with the rs7525160 C allele carriers in smokers with OR (95% CI) of 1.72 (1.15-2.79), but not in non-smokers with OR (95% CI) of 1.15 (0.81-1.65). When the interaction between smoking status and rs7525160 G > C variant was analyzed with cumulative smoking dose (pack-year). Similarly, GC or CC genotype carriers have increased risk of NSCLC among heavy smokers (pack-year ≥ 25) with OR (95% CI) of 2.01 (1.26-3.20), but not among light smokers (pack-year <25) with OR (95% CI) of 1.32 (0.81-2.16).

**Conclusion:**

*CR1* rs7525160 G > C polymorphism was associated with an increased risk of developing NSCLC in Chinese population. The association displays a manner of gene-environmental interaction between *CR1* rs7525160 tagSNP and smoking status.

## Background

The complement system plays a critical role in the process of carcinogenesis. Despite of significant research, controversial viewpoints remain on the exact relationship of complement system with cancer. Classically, the complement system fights against cancer by exerting the effects of immunosurveillance in the immunologic microenvironment of tumors [1]. Recently it was found that complement may contribute to tumor growth by a wide variety of mechanisms including dysregulation of mitogenic signaling pathways, sustained cellular proliferation, angiogenesis, insensitivity to apoptosis, invasion and migration, and escape from complement cytotoxicity [2]. This suggested complement, just like a double-edged sword, plays a dual role in carcinogenesis. In particular, component C3 and its receptors have been demonstrated to be a key link between innate and adaptive immunity [3].

Complement receptor type 1 (CR1, CD35) is a multifunctional polymorphic glycoprotein which binds to C3b fragment of C3 and to C4b with lower affinity [4,5]. CR1 belongs to the regulators of complement activation (RCA) family of proteins and is expressed in a wide spectrum of cells and involved in T-cell and B-cell mediated immune regulation [6,7]. CR1 also modulates the complement cascade activation by preventing formation of classical and alternative pathway convertases and by acting as a cofactor for factor I mediated inactivation of C3b and C4b [8,9]. It has been demonstrated that chronic inflammation can predispose to cancer development and spread [10], as a fundamental component of innate immunity, the complement cascade consists of potential proinflammatory molecules especially C3 and C5. Moreover, complement activation and abnormal expression in tumor tissues has been demonstrated [11]. Considering the important role of CR1 in complement activation, innate immunity and chronic inflammation, CR1 has emerged as a molecule of immense interest in gaining insight into the susceptibility to cancer.

*CR1* gene is located on the Chromosome 1 at the locus 1q32 [12]. Various polymorphisms have been studied including the intronic and exonic density polymorphism for their ability to alter the density of erythrocyte CR1 on the cell membranes [13-15]. There are also the molecular weight variants due to insertion-deletion polymorphisms [16]. Up to now, there have been very few studies on the association of genetic variants of CR1 with susceptibility to autoimmune and inflammatory diseases. It has been proposed that genetic variant at *CR1* gene (rs6656401) might influence the susceptibility to late-onset Alzheimer’s disease [17]. CR1 expression in Peripheral Blood Mononuclear Cells (PBMCs) may be a new biomarker for prognosis of nasopharyngeal carcinoma and a potential therapeutic target [18]. Recently, it has been indicated that *CR1* A3650G (His1208Arg) polymorphism plays a critical role in conferring genetic susceptibility to gallbladder cancer in north Indian population [19]. However, the association of genetic variants of *CR1* with risk of lung cancer remains unexplored.

Worldwide, lung cancer is the most common cancer in terms of both incidence and mortality [20]. NSCLC is the most common subtype of lung cancer and less aggressive and metastic than SCLC. Although cigarette smoking is the predominant risk factor for lung cancer, inherited genetic characteristics are presumed to account in part for this interindividual variation in lung cancer susceptibility. Recently, several genome-wide association studies have demonstrated the common genetic variations associated with susceptibility to lung cancer [21-24]. Given the involvement of the complement system in coordinating innate immunity and inflammatory response [25], further examination of the potential association between genetic variation of *CR1* genes and lung cancer is warranted.

In the current study, we conducted a case-control study to investigate the association of tag SNPs in *CR1* gene with the risk of NSCLC and effect of the interaction of gene-environment on the risk of NSCLC.

## Results

### Subject characteristics

The frequency distributions of select characteristics in cases and control subjects were shown in Table 1. The mean age (±SD) was 59.6 ± 10.5 years for the cancer patients and 57.2 ± 13.3 years for the controls. No significant difference was found in the mean age between cases and controls (*P* = 0.470). There was no significant difference in proportion of sex and smoking status between cases and controls (*P* = 0.832 and *P* = 0.321, respectively). However, there was significant difference between cases and controls when compared by pack-year smoked (*P* = 0.001). The heavy smokers (≥25 pack-year) accounted for 61.5% in cases but only 45.5% in controls, which suggested that cigarette smoking was a prominent contributor to the risk of lung cancer. Of the 470 case patients, 178 (37.9%) were diagnosed as adenocarcinoma, 238 (50.6%) as squamous cell carcinoma, and 100 (%) as other types, including large cell carcinoma (n = 49) and mixed cell carcinoma (n = 5).

**Table 1:** Distributions of select characteristics in cases and control subjects

**Variables**	** Cases (n = 470)**		** Controls (n = 470)**		
	**No**	**(%)**	**No**	**(%)**	***P***^**a**^
Sex					0.832
Male	324	68.9	328	69.8	
Female	146	31.1	142	30.2	
Age					0.470
<50	84	17.9	96	20.4	
50-59	177	37.7	187	39.8	
60-69	129	27.4	111	23.6	
≥70	80	17.0	76	16.2	
Smoking status					0.321
Non-smoker	265	56.4	281	59.8	
Smoker	205	43.6	189	40.2	
Pack-year smoked					0.001
<25	75	36.6	96	50.8	
≥25	130	63.4	93	49.2	

^a^Two-sided χ^2^ test.

### Association of *CR1* tag SNP with NSCLC risk

Total 13 selected tag SNPs of CR1 in HapMap database among Chinese population were analyzed. Except for rs9429782 polymorphism, the genotype distributions of other SNPs in controls were consistent to Hardy-Weinberg equilibrium. Therefore, we excluded the rs9429782 from further analysis. In order to screen the genetic variants that confer the susceptibility to lung cancer, 12 candidate tagSNPs were genotyped in a case-control study consisting of 470 lung cancer patients and 470 cancer-free controls as shown in Table 2. Importantly, genotype frequency of one intronic SNP (rs7525160 G > C) in cases was found to be significantly different from those of controls (χ^2^ = 6.339, *P*=0.042). Further multivariate regression model with adjustment for age, gender, and smoking status was used to assess the association between rs7525160 G > C polymorphism and the risk of NSCLC. The results indicated that the rs7525160 CC genotype was associated with an increased risk of developing NSCLC with OR (95% CI) of 1.52 (1.02-2.28) compared with the GG genotype. Other tagSNPs of *CR1* were not significantly associated with the risk of NSCLC in our study population (*P* >0.05).

**Table 2:** Genotype frequencies of CRI among cases and controls and their association with non-small cell lung cancers

**CRI Genotypes**	** Controls (n = 470)**		** Cases (n = 470)**		***OR *** (95% *CI*)^*^	***P***
	**No**	**(%)**	**No**	**(%)**		
rs7525160						
GG	176	37.5	139	29.6	1.00 (ref.)	
CG	228	48.5	256	54.5	1.38 (1.04-1.85)	0.041
CC	66	14.0	75	15.9	1.52 (1.02-2.28)	0.028
rs3886100						
GG	117	24.9	105	22.4	1.00 (ref.)	
AG	223	47.4	253	53.8	1.33 (0.97-1.81)	0.078
AA	130	27.7	112	23.8	1.06 (0.73-1.54)	0.755
rs11118167						
TT	348	74.1	353	75.1	1.00 (ref.)	
CT	111	23.6	102	21.7	0.89 (0.65-1.21)	0.457
CC	11	2.3	15	3.2	1.35 (0.61-3.01)	0.461
rs9429782						
GG	250	53.2	261	55.5	1.00 (ref.)	
GT	220	46.8	209	44.5	0.89 (0.69-1.16)	0.388
rs10494885						
CC	178	37.9	164	34.9	1.00 (ref.)	
CT	224	47.6	232	49.4	1.11 (0.83-1.47)	0.490
TT	68	14.5	74	15.7	1.20 (0.81-1.78)	0.365
rs7542544						
CC	128	27.2	108	23.0	1.00 (ref.)	
AC	223	47.5	252	53.6	1.21 (0.88-1.67)	0.239
AA	119	25.3	110	23.4	0.90 (0.62-1.30)	0.897
rs6691117						
AA	324	68.9	327	69.6	1.00 (ref.)	
AG	131	27.9	128	27.2	0.98 (0.73-1.31)	0.888
GG	15	3.2	15	3.2	0.96 (0.46-2.02)	0.923
rs6656401						
GG	436	92.8	447	95.1	1.00 (ref.)	
AG	34	7.2	23	4.9	0.68 (0.39-1.18)	0.174
AA	0	0.0	0	0.0	NC^§^	
rs2296160						
CC	185	39.4	194	41.3	1.00 (ref.)	
CT	226	48.1	220	46.8	0.91 (0.69-1.21)	0.521
TT	59	12.5	56	11.9	0.90 (0.59-1.37)	0.606
rs9429942						
TT	452	96.2	457	97.2	1.00 (ref.)	
CT	18	3.8	13	2.8	0.77 (0.37-1.61)	0.482
CC	0	0.0	0	0.0	NC^§^	
rs4844600						
GG	171	36.4	179	38.1	1.00 (ref.)	
AG	230	48.9	228	48.5	0.92 (0.70-1.22)	0.571
AA	69	14.7	63	13.4	0.87 (0.58-1.31)	0.513
rs3818361						
CC	187	39.8	188	40.0	1.00 (ref.)	
CT	224	47.7	224	47.7	0.98 (0.74-1.29)	0.868
TT	59	12.5	58	12.3	0.96 (0.63-1.46)	0.848
rs17048010						
TT	301	64.0	286	60.8	1.00 (ref.)	
CT	154	32.8	164	34.9	1.09 (0.82-1.43)	0.556
CC	15	3.2	20	4.3	1.40 (0.70-2.79)	0.343

^*^Adjusted by age, sex and smoking status; ^§^NC, not calculated.

**Table 3:** Summary of MDR gene-gene interaction results

**Models**	**Training bal. acc. (%)**	**Testing bal. acc. (%)**	***P *** **value**	**Cross-validation consistency**
rs7525160	54.03	50.53	0.828	7/10
rs4844600, rs10494885	55.45	49.32	0.989	3/10
rs4844600, rs10494885, rs7525160	57.60	48.48	0.623	6/10

Generalized Multifactor Dimensionality Reduction (GMDR) was used to evaluate gene-gene interaction. The summary of gene-gene interaction models is listed in Table 3. The SNP rs7525160 in CR1 had the highest testing balanced accuracy among 12 SNPs. The three-way interaction model among rs4844600, rs10494885 and rs7525160 showed high testing balance accuracy and cross validation consistency, but the testing balanced accuracy was lower than the two-way gene-gene interaction in NSCLC. For each model, the interaction was not significant (*P* > 0.05).

**Table 4:** Risk of CR1 genotypes with NSCLC by smoking status

**Smoking status**	**CR1 genotype**					
	GG^*^	**OR (95% CI)**^**§**^	***P *** **value**	CG + CC^*^	**OR (95% CI)**^**§**^	***P *** **value**
Non-smoker	84/99	1.00 (reference)		181/182	1.15 (0.81-1.65)	0.440
Smoker	55/77	0.86 (0.54-1.38)	0.528	150/112	1.72 (1.15-2.59)	0.009
<25 pack-years	19/41	0.59 (0.31-1.10)	0.099	56/55	1.32 (0.81-2.61)	0.266
≥25 pack-years	36/36	1.18 (0.67-2.08)	0.562	94/57	2.01 (1.26-3.20)	0.003

^*^Number of cases/number of controls.

^§^Data were calculated by logistic regression and adjusted for age and gender.

### Interaction of *CR1* SNP with smoking

Cigarette smoking is a well-known risk for lung cancer, so stratification by smoking status was performed to investigate the association of rs7525160 G > C variant with the risk of NSCLC. As shown in Table 4, the risk of NSCLC was associated with the rs7525160 C allele carriers in smokers with OR (95% CI) of 1.72 (1.15-2.59), but not in non-smokers with OR (95% CI) of 1.15 (0.81-1.65), suggesting that the *CR1* rs7525160 G > C polymorphism is a smoking-modifying risk factor for susceptibility to NSCLC. When the interaction between smoking status and rs7525160 G > C variant was analyzed with cumulative smoking dose (pack-year), consistently, GC or CC genotype carriers have increased risk of NSCLC among heavy smokers (pack-year ≥ 25) with OR (95% CI) of 2.01 (1.26-3.20), but not among light smokers (pack-year <25) with OR (95% CI) of 1.32 (0.81-2.16). The *P* value for heterogeneity of the stratification analysis by smoking status is 0.015. However, the P value for interaction between rs7525160 polymorphism and smoking is 0.172 and the power for the interaction is 0.49.

## Discussion

The chronic airway inflammation and dysfunctional immune system might promote pulmonary carcinogenesis. Implicated in the immune and inflammatory responses, the complement cascade plays a pivotal role in the development of cancer. Thus, it is likely that the genetic variants of CR1 in the complement system confer the susceptibility to lung cancer. In this study, we have for the first time demonstrated that one intronic SNP (rs7525160 G > C) out of 13 tag SNPs of CR1 was associated with the risk of NSCLC in Chinese population. Notably, the rs7525160 CC genotype was associated with an increased risk of developing NSCLC (OR = 1.52, 95% CI = 1.02-2.28; *P* = 0.028) compared with the GG genotype. MDR analysis also showed that there was no gene-gene interaction among 12 tag SNPs in CR1 gene. Moreover, the risk of NSCLC was associated with the rs7525160 C allele carriers in smokers with OR (95% CI) of 1.72 (1.15-2.59), but not in non-smokers with OR (95% CI) of 1.15 (0.81-1.65), indicating this SNP is a smoking-modifying risk factor for susceptibility to NSCLC. To the best of our knowledge, this study shed new insight into the interplay of genetic variation of CR1 with lung cancer risk. More importantly, it highlights the potential gene-environmental interaction influences the susceptibility to lung cancer.

The complement system has been proposed to get involved in innate immunity with the ability to “complement” antibody-mediated elimination of immune complex and foreign pathogens [26]. Upon complement activation, the biologically active peptides C5a and C3a elicit a lot of pro-inflammatory effects and could be closely associated with tumorigenesis [27]. Complement proteins play a dual role in the tumor microenvironment. On one hand, they exert a defensive effect against tumor through complement or antibody-dependent cytotoxicity [1,28]. On the other hand, they may escape from immunosurveillance and facilitate carcinogenesis [2]. Specifically, a number of experimental evidence has suggested an association between complement activation and tumor growth [29,30], which provides a strong biologically link between the abnormal expression and activity of complement cascade and carcinogenesis.

Till now, a few studies have been carried out to demonstrate the association of genetic variants in complement proteins with susceptibility to cancer. A significant association of CR2 SNP (rs3813946) with the development of nasopharyngeal carcinoma was indicated in Cantonese population [31], and the genetic variations of complement system genes C5 and C9 plays a potential role in susceptibility to non-Hodgkin lymphoma (NHL) [32]. Recently, it has been shown that complement factor H Y402H polymorphism interact with cigarette smoking to confer the susceptibility to lung cancer [33]. Furthermore, it has been indicated that *CR1* A3650G (His1208Arg) polymorphism plays a critical role in conferring genetic susceptibility to gallbladder cancer in north Indian population [19]. However, whether the genetic variants of *CR1* are related to the risk of lung cancer remains unknown. In this case-control study, we found an intronic SNP (rs7525160 G > C) with CC genotype was significantly associated with an increased risk of NSCLC. Consistently, our results were in accordance with the study that genetic polymorphisms in innate immunity genes may play a role in the carcinogenesis of lung cancer [34]. It is likely that some genetic variations in strong link disequilibrium with this intronic SNP (rs7525160 G > C) are functional, which provides a new insight into the hallmarks in susceptibility to lung cancer and further functional experiments are warranted to address the proposal.

Functionally, human CR1 exists on the surface of almost all peripheral blood cells and plays a key role in immune complex clearance and complement inhibition at the cell surface by binding to activated products C3b and C4b [4,35]. CR1 also possesses cofactor activity for the serum protease factor I and is thus involved in the generation of further fragments of C3/4b with the activation of complement cascade and the cellular immune response [4]. In our study, the association of *CR1* polymorphism with lung cancer is biologically plausible in that the intronic polymorphism could affect the density of CR1 molecules on the cell surface, thereby contributing to autoimmune disorders and neoplasm.

Tobacco smoking is an established risk factor for susceptibility to lung cancer. However, not all people who suffer from lung cancer are smokers. Lung cancer in non-smokers can be induced by second hand smoke, air pollutants and diesel exhaust [36-39]. Our present data showed significant difference of pack-year smoked, but not smoking status between NSCLC cases and controls, which suggested the important role of other environmental factors in the development of NSCLC. Tobacco could induce chronic and sustained inflammation in lung microenvironment, contributing to pulmonary carcinogenesis in smokers [40]. Support also comes from the epidemiologic data regarding inflammation and lung cancer [41]. CR1, an important molecule implicated in immunity and inflammation, could protect the host from invasion of exogenous chemicals derived from cigarette smoking. Genetic variant of CR1 could alter gene function and result in deregulation of the inflammatory and immune responses, thereby, modulating the susceptibility to lung cancer. More importantly, we observed a potential interaction of this SNP (rs7525160 G > C) with smoking status, suggesting the gene-environmental interaction plays a prominent role in the susceptibility to lung cancer. Our present study has its limitation. Our patients may not be representative of total NSCLC patients at large because they were recruited from only one hospital. In addition, due to the relatively small sample size, further case-control studies are still needed to replicate and extend our findings.

## Conclusion

We conducted a case-control study in Chinese subjects and found an intronic SNP (rs7525160 G > C) of *CR1* was significantly associated with lung cancer risk. To the best of our knowledge, this study provides the first evidence that genetic variant of *CR1* (rs7525160 G > C) was a smoking-modifying contributor to the development of lung cancer.

## Methods

### Study subjects

This case-control study consisted of 470 patients with histopathologically confirmed NSCLC and 470 cancer-free controls. All subjects were genetic unrelated ethnic Han Chinese. Patients were recruited between January 2008 and December 2012 at Tangshan Gongren Hospital (Tangshan, China). There were no age, gender or stage restrictions, however, patients with previous malignancy or metastasized cancer from other organs were excluded. The response rate for patients was 94%. The controls were randomly selected from a pool of a cancer-free population from a nutritional survey conducted in the same region. The selection criteria for control subjects included: i) no individual history of cancer; ii) frequency matched to cases according to gender, age (±5 years); iii) the residential region; and iv) the time period for blood sample collection. At recruitment, informed consent was obtained from each subject, and each participant was then interviewed to collect detailed information on demographic characteristics. This study was approved by the institutional review board of Hebei United University.

### Tag SNPs selection and genotyping

Based on the Chinese population data from HapMap database, we used Haploview 4.2 program to select candidate tag SNPs with an *r*^*2*^ threshold of 0.80 and minor allele frequency (MAF) greater than 1%. Furthermore, we also added two potential functional polymorphisms, rs9429942 and rs6691117 [42,43]. Therefore, we included 13 SNPs in our study, which represents common genetic variants in Chinese population.

Genotyping was performed at Bomiao Tech (Beijing, China) using iPlex Gold Genotyping Asssy and Sequenom MassArray (Sequenom, San Diego, CA, USA). Sequenom’s MassArray Designer was used to design PCR and extension primers for each SNP. Primer information for selected tag SNPs was listed in Table 5.

**Table 5:** Primers used in this study

**SNP_ID**	**Alleles**	**1st-PCR primer sequences**	**2nd-PCR primer sequences**	**UEP sequences**
rs7525160	G/C	ACGTTGGATGCAAAATCAAGGTTTAAAGTC	ACGTTGGATGTTCTGACATGTACTGCCTGC	CCCTGTTGCCTGGGTTTTTCT
rs3886100	G/A	ACGTTGGATGGGCCTCAGATCCTCAAAATC	ACGTTGGATGTGAGCTGTTTCAGCCAAGAG	GAGCCAAGAGGACACTTAG
rs11118167	T/C	ACGTTGGATGATGTGTGTAGTCACTTAGCC	ACGTTGGATGATAATGGCAGATTTAAGGGC	CAATGATAAATGAATACTGTGTTCTATC
rs9429782	G/T	ACGTTGGATGACACGCGGGATCCATCGGAA	ACGTTGGATGAACGAGTTTCGCTGGCAGAG	GGTGCAGCAGCAGAG
rs10494885	C/T	ACGTTGGATGGTGTAATGCCACAGACATGC	ACGTTGGATGCCAGCCAACTGACCTTTATG	CTTCTGATTTTCTTTCCTGTTAC
rs7542544	C/A	ACGTTGGATGGCTAAGAGCCATTAGTGTGC	ACGTTGGATGAACGTGGTGGTGCCCAAACA	CCATGACCCCAAAGC
rs6691117	A/G	ACGTTGGATGAGAGTACCAGGAAACAGGAG	ACGTTGGATGACCCTACCATGACAAACCCG	CCGGGCTGACATCTAAATCTGA
rs6656401	G/A	ACGTTGGATGAAAGGACACACACAGAGGAG	ACGTTGGATGCGTTGATGTTCCTTGGCTTG	CTCTGTCTCCATCTTCTC
rs2296160	C/T	ACGTTGGATGCCAGAATTCCTCAGCAAAAC	ACGTTGGATGCCAGAGTGATGTTTTGTGAC	CGTGCCTTTTGTCTTCCTTTTAGGT
rs9429942	T/C	ACGTTGGATGTACATGTGCACAACGTGCAG	ACGTTGGATGAAGGACGAGTTAATGGGTGC	GGGAACGTCGCACATGTAT
rs4844600	G/A	ACGTTGGATGGAATGGCTTCCATTTGCCAG	ACGTTGGATGGGGCGGCATTCATAGTTCAG	CCCAATGGGAAACTCAAA
rs3818361	C/T	ACGTTGGATGTGGAAAGGACAGTTCCAGAG	ACGTTGGATGTTTTAAGCCCTCTGGTAAGC	TAATCCCTCTGGTAAGCATAAGATATA
rs17048010	T/C	ACGTTGGATGTTTCAAGGCTGCTCCTTGTT	ACGTTGGATGCCCAGTCTATGGAGTTTCTG	AGACTGAGACAGTTGGT

### Statistical analysis

We used Chi-square test to examine the differences in the distributions of demographic characteristics, and genotype frequencies between cases and controls. The NSCLC risk associated with *CR1* tag SNPs was estimated as odds ratios (OR) and 95% confidence intervals (CI) computed by logistic regression model adjusted for age, gender, and smoking status where it was appropriate. Smokers were considered current smokers if they smoked up to 1 year before the date of cancer diagnosis for NSCLC patients or before the date of the interview for controls. The number of pack-years smoked was determined as an indication of cumulative cigarette-dose level [pack-year = (cigarettes per day/20) × (years smoked)]. Light and heavy smokers were categorized by using the 50th percentile pack-year value of the controls as the cut points (i.e., ≤25 and >25 pack-years). All statistical tests were 2 sided with *P*< 0.05 as the significant level. Statistical analyses were done using SPSS (version 16.0, SPSS Inc, Chicago, IL). Gene-gene and gene-smoking interactions were analyzed by open-resource GMDR software package (version 0.9) and Quanto (http://www.hydra.usc.edu/gxe) [44,45].

## Abbreviations

CR1: Complement receptor 1; OR: Odds ratio; CI: Confidence interval; SNP: Single nucleotide polymorphism.

## Competing interests

The authors declare no competing financial interest.

## Authors’ contributions

XY drafted the article. XY and JR conducted genotyping of CR1. JL, ZZ and LC collected clinical data and analyzed the data. XZ contributed the research plan and approved the data. All authors read and approved the final manuscript.

## References

[B1] Gelderman KA Tomlinson S Ross GD Gorter A Complement function in mAb-mediated cancer immunotherapy Trends Immunol 2004 25 158 164 10.1016/j.it.2004.01.008 15036044

[B2] Rutkowski MJ Sughrue ME Kane AJ Mills SA Parsa AT Cancer and the complement cascade Mol Cancer Res 2010 8 1453 1465 10.1158/1541-7786.MCR-10-0225 20870736

[B3] Liu D Niu ZX The structure, genetic polymorphisms, expression and biological functions of complement receptor type 1 (CR1/CD35) Immunopharmacol Immunotoxicol 2009 31 524 535 10.3109/08923970902845768 19874218

[B4] Ahearn JM Fearon DT Structure and function of the complement receptors, CR1 (CD35) and CR2 (CD21) Adv Immunol 1989 46 183 219 2551147 10.1016/s0065-2776(08)60654-9

[B5] Tas SW Klickstein LB Barbashov SF Nicholson-Weller A C1q and C4b bind simultaneously to CR1 and additively support erythrocyte adhesion J Immunol 1999 163 5056 5063 10528211

[B6] Wagner C Ochmann C Schoels M Giese T Stegmaier S Richter R Hug F Hansch GM The complement receptor 1, CR1 (CD35), mediates inhibitory signals in human T-lymphocytes Mol Immunol 2006 43 643 651 10.1016/j.molimm.2005.04.006 16360013

[B7] Jozsi M Prechl J Bajtay Z Erdei A Complement receptor type 1 (CD35) mediates inhibitory signals in human B lymphocytes J Immunol 2002 168 2782 2788 11884446 10.4049/jimmunol.168.6.2782

[B8] Rochowiak A Niemir ZI The structure and role of CR1 complement receptor in physiology Pol Merkur Lekarski 2010 28 79 83 20369732

[B9] Ross GD Lambris JD Cain JA Newman SL Generation of three different fragments of bound C3 with purified factor I or serum. I. Requirements for factor H vs CR1 cofactor activity J Immunol 1982 129 2051 2060 6214588

[B10] Coussens LM Werb Z Inflammation and cancer Nature 2002 420 860 867 10.1038/nature01322 12490959 PMC2803035

[B11] Jurianz K Ziegler S Garcia-Schuler H Kraus S Bohana-Kashtan O Fishelson Z Kirschfink M Complement resistance of tumor cells: basal and induced mechanisms Mol Immunol 1999 36 929 939 10.1016/S0161-5890(99)00115-7 10698347

[B12] Weis JH Morton CC Bruns GA Weis JJ Klickstein LB Wong WW Fearon DT A complement receptor locus: genes encoding C3b/C4b receptor and C3d/Epstein-Barr virus receptor map to 1q32 J Immunol 1987 138 312 315 3782802

[B13] Wilson JG Wong WW Murphy EE 3rd Schur PH Fearon DT Deficiency of the C3b/C4b receptor (CR1) of erythrocytes in systemic lupus erythematosus: analysis of the stability of the defect and of a restriction fragment length polymorphism of the CR1 gene J Immunol 1987 138 2708 2710 2881967

[B14] Xiang L Rundles JR Hamilton DR Wilson JG Quantitative alleles of CR1: coding sequence analysis and comparison of haplotypes in two ethnic groups J Immunol 1999 163 4939 4945 10528197

[B15] Birmingham DJ Chen W Liang G Schmitt HC Gavit K Nagaraja HN A CR1 polymorphism associated with constitutive erythrocyte CR1 levels affects binding to C4b but not C3b Immunology 2003 108 531 538 10.1046/j.1365-2567.2003.01579.x 12667215 PMC1782929

[B16] Holers VM Chaplin DD Leykam JF Gruner BA Kumar V Atkinson JP Human complement C3b/C4b receptor (CR1) mRNA polymorphism that correlates with the CR1 allelic molecular weight polymorphism Proc Natl Acad Sci U S A 1987 84 2459 2463 10.1073/pnas.84.8.2459 3031685 PMC304671

[B17] Zhang Q Yu JT Zhu QX Zhang W Wu ZC Miao D Tan L Complement receptor 1 polymorphisms and risk of late-onset Alzheimer’s disease Brain Res 2010 1348 216 221 20558149 10.1016/j.brainres.2010.06.018

[B18] He JR Xi J Ren ZF Qin H Zhang Y Zeng YX Mo HY Jia WH Complement receptor 1 expression in peripheral blood mononuclear cells and the association with clinicopathological features and prognosis of nasopharyngeal carcinoma Asian Pac J Cancer Prev 2012 13 6527 6531 10.7314/APJCP.2012.13.12.6527 23464487

[B19] Srivastava A Mittal B Complement receptor 1 (A3650G RsaI and intron 27 HindIII) polymorphisms and risk of gallbladder cancer in north Indian population Scand J Immunol 2009 70 614 620 10.1111/j.1365-3083.2009.02329.x 19906204

[B20] Ferreira CG Lung cancer in developing countries Am Soc Clin Oncol Educ Book 2013 327 331 PMID:23714537 23714537 10.14694/EdBook_AM.2013.33.327

[B21] Amos CI Wu X Broderick P Gorlov IP Gu J Eisen T Dong Q Zhang Q Gu X Vijayakrishnan J Sullivan K Matakidou A Wang Y Mills G Doheny K Tsai YY Chen WV Shete S Spitz MR Houlston RS Genome-wide association scan of tag SNPs identifies a susceptibility locus for lung cancer at 15q25.1 Nat Genet 2008 40 616 622 10.1038/ng.109 18385676 PMC2713680

[B22] Hung RJ McKay JD Gaborieau V Boffetta P Hashibe M Zaridze D Mukeria A Szeszenia-Dabrowska N Lissowska J Rudnai P Fabianova E Mates D Bencko V Foretova L Janout V Chen C Goodman G Field JK Liloglou T Xinarianos G Cassidy A McLaughlin J Liu G Narod S Krokan HE Skorpen F Elvestad MB Hveem K Vatten L Linseisen J A susceptibility locus for lung cancer maps to nicotinic acetylcholine receptor subunit genes on 15q25 Nature 2008 452 633 637 10.1038/nature06885 18385738

[B23] Wang Y Broderick P Webb E Wu X Vijayakrishnan J Matakidou A Qureshi M Dong Q Gu X Chen WV Spitz MR Eisen T Amos CI Houlston RS Common 5p15.33 and 6p21.33 variants influence lung cancer risk Nat Genet 2008 40 1407 1409 10.1038/ng.273 18978787 PMC2695928

[B24] McKay JD Hung RJ Gaborieau V Boffetta P Chabrier A Byrnes G Zaridze D Mukeria A Szeszenia-Dabrowska N Lissowska J Rudnai P Fabianova E Mates D Bencko V Foretova L Janout V McLaughlin J Shepherd F Montpetit A Narod S Krokan HE Skorpen F Elvestad MB Vatten L Njølstad I Axelsson T Chen C Goodman G Barnett M Loomis MM Lung cancer susceptibility locus at 5p15.33 Nat Genet 2008 40 1404 1406 10.1038/ng.254 18978790 PMC2748187

[B25] Markiewski MM Lambris JD The role of complement in inflammatory diseases from behind the scenes into the spotlight Am J Pathol 2007 171 715 727 10.2353/ajpath.2007.070166 17640961 PMC1959484

[B26] Ricklin D Lambris JD Complement-targeted therapeutics Nat Biotechnol 2007 25 1265 1275 10.1038/nbt1342 17989689 PMC2966895

[B27] Hugli TE Biochemistry and biology of anaphylatoxins Complement 1986 3 111 127 3542363 10.1159/000467889

[B28] Ostrand-Rosenberg S Cancer and complement Nat Biotechnol 2008 26 1348 1349 10.1038/nbt1208-1348 19060872 PMC2800820

[B29] Markiewski MM DeAngelis RA Benencia F Ricklin-Lichtsteiner SK Koutoulaki A Gerard C Coukos G Lambris JD Modulation of the antitumor immune response by complement Nat Immunol 2008 9 1225 1235 10.1038/ni.1655 18820683 PMC2678913

[B30] Markiewski MM Lambris JD Is complement good or bad for cancer patients? A new perspective on an old dilemma Trends Immunol 2009 30 286 292 10.1016/j.it.2009.04.002 19428302 PMC2704572

[B31] Fan Q He JF Wang QR Cai HB Sun XG Zhou XX Qin HD Shugart YY Jia WH Functional polymorphism in the 5′-UTR of CR2 is associated with susceptibility to nasopharyngeal carcinoma Oncol Rep 2013 30 11 16 23612877 10.3892/or.2013.2421 PMC3729234

[B32] Cerhan JR Novak AJ Fredericksen ZS Wang AH Liebow M Call TG Dogan A Witzig TE Ansell SM Habermann TM Kay NE Slager SL Risk of non-Hodgkin lymphoma in association with germline variation in complement genes Br J Haematol 2009 145 614 623 10.1111/j.1365-2141.2009.07675.x 19344414 PMC2820509

[B33] Zhang Z Yu D Yuan J Guo Y Wang H Zhang X Cigarette smoking strongly modifies the association of complement factor H variant and the risk of lung cancer Cancer Epidemiol 2012 36 e111 e115 10.1016/j.canep.2011.11.004 22197220

[B34] Shen M Vermeulen R Rajaraman P Menashe I He X Chapman RS Yeager M Thomas G Burdett L Hutchinson A Yuenger J Chanock S Lan Q Polymorphisms in innate immunity genes and lung cancer risk in Xuanwei, China Environ Mol Mutagen 2009 50 285 290 10.1002/em.20452 19170196 PMC2666781

[B35] Fearon DT Identification of the membrane glycoprotein that is the C3b receptor of the human erythrocyte, polymorphonuclear leukocyte, B lymphocyte, and monocyte J Exp Med 1980 152 20 30 10.1084/jem.152.1.20 6967510 PMC2185895

[B36] Besaratinia A Pfeifer GP Second-hand smoke and human lung cancer Lancet Oncol 2008 9 657 666 10.1016/S1470-2045(08)70172-4 18598930 PMC2740739

[B37] Coyle YM Minahjuddin AT Hynan LS Minna JD An ecological study of the association of metal air pollutants with lung cancer incidence in Texas J Thorac Oncol 2006 1 654 661 10.1097/01243894-200609000-00009 17409932

[B38] Garshick E Laden F Hart JE Rosner B Smith TJ Dockery DW Speizer FE Lung cancer in railroad workers exposed to diesel exhaust Environ Health Perspect 2004 112 1539 1543 10.1289/ehp.7195 15531439 PMC1247618

[B39] Brennan P Buffler PA Reynolds P Wu AH Wichmann HE Agudo A Pershagen G Jockel KH Benhamou S Greenberg RS Merletti F Winck C Fontham ET Kreuzer M Darby SC Forastiere F Simonato L Boffetta P Secondhand smoke exposure in adulthood and risk of lung cancer among never smokers: a pooled analysis of two large studies Int J Cancer 2004 109 125 131 10.1002/ijc.11682 14735478

[B40] Lee G Walser TC Dubinett SM Chronic inflammation, chronic obstructive pulmonary disease, and lung cancer Curr Opin Pulm Med 2009 15 303 307 10.1097/MCP.0b013e32832c975a 19417670

[B41] Engels EA Inflammation in the development of lung cancer: epidemiological evidence Expert Rev Anticancer Ther 2008 8 605 615 10.1586/14737140.8.4.605 18402527

[B42] Kullo IJ Ding K Shameer K McCarty CA Jarvik GP Denny JC Ritchie MD Ye Z Crosslin DR Chisholm RL Manolio TA Chute CG Complement receptor 1 gene variants are associated with erythrocyte sedimentation rate Am J Hum Genet 2011 89 131 138 10.1016/j.ajhg.2011.05.019 21700265 PMC3135803

[B43] Teeranaipong P Ohashi J Patarapotikul J Kimura R Nuchnoi P Hananantachai H Naka I Putaporntip C Jongwutiwes S Tokunaga K A functional single-nucleotide polymorphism in the CR1 promoter region contributes to protection against cerebral malaria J Infect Dis 2008 198 1880 1891 10.1086/593338 18954261

[B44] Chen GB Xu Y Xu HM Li MD Zhu J Lou XY Practical and theoretical considerations in study design for detecting gene-gene interactions using MDR and GMDR approaches PLoS One 2011 6 e16981 10.1371/journal.pone.0016981 21386969 PMC3046176

[B45] Lou XY Chen GB Yan L Ma JZ Zhu J Elston RC Li MD A generalized combinatorial approach for detecting gene-by-gene and gene-by-environment interactions with application to nicotine dependence Am J Hum Genet 2007 80 1125 1137 10.1086/518312 17503330 PMC1867100

